# A Paper-Based Assay for the Determination of Total Antioxidant Capacity in Human Serum Samples

**DOI:** 10.3390/bios14110559

**Published:** 2024-11-18

**Authors:** Michelle T. Tran, Sophia V. Gomez, Vera Alenicheva, Vincent T. Remcho

**Affiliations:** Department of Chemistry, Oregon State University, Corvallis, OR 97331, USA; tranmic@oregonstate.edu (M.T.T.); gomezso@oregonstate.edu (S.V.G.); alenichv@oregonstate.edu (V.A.)

**Keywords:** Trolox equivalent antioxidant capacity (TEAC), 2,2′-azinobis(3-ethylbenzothiazoline-6-sulfonic acid) (ABTS), microfluidic paper-based analytical device (µPAD), total antioxidant capacity (TAC), antioxidant

## Abstract

Determining the total antioxidant capacity (TAC) of biological samples is a valuable approach to measuring health status under oxidative stress conditions, such as infertility and type 2 diabetes. The Trolox equivalent antioxidant capacity (TEAC) assay is the most common approach to evaluating TAC in biological matrices. This assay is typically performed in clinical settings on a microtiter plate using a plate reader. However, the instrumentation and expertise requirements, and the resulting delay in the reporting of assay outcomes, make solution-based TEAC assays impractical for point-of-care or at-home testing, where individuals may want to monitor their health status during treatment. This work introduces the first microfluidic paper-based analytical device (µPAD) that measures TAC in human serum using TEAC assay chemistry. TAC was determined through a colorimetric image analysis of the degree of decolorization of 2,2′-azinobis(3-ethylbenzothiazoline-6-sulfonic acid) radical cations (ABTS^●+^) by serum antioxidants. The µPAD showed a linear response to Trolox, ranging from 0.44 to 2.4 mM, (r  =  0.999). The performance of paper-based TEAC assays was validated through direct comparison to solution-based TEAC assays. There was a 0.04 mM difference in TAC values between the two platforms, well within one standard deviation of a standard solution-based assay conducted on an aliquot of the same serum sample (±0.25 mM). The µPAD had a limit of detection (LOD) of 0.20 mM, well below the TAC of normal human serum. The results suggest that the proposed device can be used for biological TAC determination and expands the field of TAC analysis in point-of-care health monitoring.

## 1. Introduction

The measurement of total antioxidant capacity (TAC) in biological fluids is a useful tool for investigating conditions related to oxidative stress. Infertility and metabolic syndrome are two commonly investigated conditions in which changes in TAC are observed [1,2,3,4,5,6,7,8]. TAC analyses of biological samples also have implications for the monitoring of other diseases associated with oxidative stress, including cardiovascular diseases [9,10,11,12], type 2 diabetes [13,14,15,16], major depressive disorder [17,18,19,20], chronic hepatitis C [21,22,23,24], and inflammatory bowel diseases [25,26,27,28,29,30].

Multiple assays have been developed to measure TAC, including an oxygen radical absorbance capacity (ORAC) assay, a 2,2-di(4-*tert*-octylphenyl)-1-picrylhydrazyl (DPPH) assay, and a ferric ion-reducing antioxidant power (FRAP) assay [31,32]. Each assay utilizes a distinct set of probes, substrates, and chemical reactions, making individual assays suitable for specific sample matrices or solvents. Despite the differences in assay components, all the aforementioned assays are based on chemical reactions that involve hydrogen transfer and/or electron transfer by antioxidants to free radicals or an oxidant [33]. This process leads to a color change that can be quantified by monitoring absorbance at a specific wavelength via spectrophotometry or fluorometry. The TAC of the sample is correlated to the kinetics or endpoint absorbance measurement.

When determining the TAC of biological samples, the assay must be appropriate for the analysis of both hydrophilic and lipophilic antioxidants. For this reason, the most common analytical strategy used to determine TAC in biological samples is the Trolox equivalent antioxidant capacity (TEAC) assay. This assay was first introduced by Miller and is based upon the inhibition of 2,2′-Azino-bis(3-ethylbenzothiazoline-6-sulfonic acid radical cations (ABTS^●+^) through antioxidant activity [34]. In a TEAC assay, a strong oxidizing agent, such as potassium persulfate (K_2_S_2_O_8_), oxidizes ABTS to form ABTS^●+^. The formation of ABTS^●+^ causes a visible change in color intensity and strong absorption maxima at 415 and 734 nm [35]. The reduction of ABTS^●+^ to ABTS via hydrogen-donating or electron-transferring Trolox standards or antioxidants present in the sample results in decolorization of the solution. The degree of decolorization is dependent on the concentration of antioxidant present, which is expressed in units of Trolox equivalents; thus, the terms “TEAC value” and “TAC value” are often used interchangeably.

The gold-standard approach to determining TAC is through solution-based assays. This process is typically carried out in clinical settings through a solution-based assay conducted on a microtiter plate for spectroscopic measurements. The most apparent disadvantage of TEAC assays is that the preparation of reagents for the assay is time-consuming, as the complete formation of ABTS^●+^ requires an overnight incubation period of 12–16 h. The resulting solution has a shelf life, when stored at 4 °C and protected from light, of 48 h. This approach is also limited in its implementation in point-of-care settings due to personnel, instrumentation, and time constraints [36]. The at-home monitoring of TAC may be pertinent in conditions such as infertility or major depressive disorder where individuals want to monitor their health status in response to a treatment over time. In these settings, a method that can easily be performed by the end-user and yields rapid results is preferred. One approach that satisfies these requirements is the adaptation of TAC assays for paper-based microfluidic analytical devices (µPAD).

Thus far, no work has been published on the development and application of TEAC-based µPADs to evaluate human serum samples, despite the prevalence of solution-based TEAC assays for evaluating biological samples. Paper-based assays have previously been developed to measure antioxidant levels in a variety of food and biological samples, including beverages, plant extracts, and vegetables [37,38,39,40]. Notably, Sun and Johnson introduced a µPAD to measure TAC in blood from murine models [41]. Their analytical device employed FRAP assay chemistry, which measures antioxidant capacity through the reduction of Fe^3+^ to Fe^2+^ rather than through a reduction in ABTS^●+^. This development is significant, but also has room for improvement. One point to consider is that the contribution of thiols from serum proteins to the TAC is only 10%, as determined via FRAP assays, because of their low reactivity with ferric ions [42]. In comparison, 30% of TAC is associated with thiol-containing proteins in the serum for TEAC assays. The use of FRAP assays may therefore be inappropriate for conditions in which serum thiol content is known to decrease, such as in coronary artery disease. Despite the prevalence of the TEAC assays being used for the TAC determination of serum samples, portable devices utilizing TEAC assay chemistry to determine TAC in blood serum have yet to be demonstrated.

This work fills that void and introduces a µPAD TEAC assay compatible with human serum samples that can determine TAC relatively quickly. The µPADs were fabricated using selective laser ablation on parafilm-backed paper to pattern up to 30 test zones. Test zones were treated with TEAC assay reagents ABTS and K_2_S_2_O_8_ at optimized concentrations of 20 mM and 10 mM, respectively. The demonstrated working range of the device was 0.44–2.40 mM (R^2^ = 0.99). The devices were successfully used to measure the TAC of human serum samples with TAC levels within one standard deviation (±0.24 mM) of the values acquired from standard, solution-based TEAC assays. The device developed, as described in this work, has a demonstrated stability of at least one day after preparation, with or without the application of samples, highlighting its potential use as an alternative to TAC monitoring using time-limited spectroscopic techniques.

## 2. Materials and Methods

### 2.1. Reagents and Materials

2,2′-Azino-bis(3-ethylbenzothiazoline-6-sulfonic acid) diammonium salt (ABTS) was purchased from Alfa Aesar, Ward Hill, MA, USA. Trolox was purchased from Acros Organics, Fairlawn, NJ, USA and K_2_S_2_O_8_ was purchased from Fisher Chemical, Pittsburg, PA, USA. Whatman grade 1 filter paper was purchased from GE Healthcare, Buckinghamshire, UK. Parafilm was purchased from Pechiney Plastic Packaging Company, Akron, OH, USA. Ultrapure water (>18 MΩ cm) was used for the preparation of all aqueous solutions (EMD Millipore, Burlington, MA, USA). A solution of 1X phosphate-buffered saline (PBS) pH 7.4 was used to prepare Trolox standards and dilutions of human serum. Stock solutions of Trolox, ABTS, and K_2_S_2_O_8_ were stored at 4 °C and used within one week. All dilutions were prepared on the day of use. Normal human serum was purchased from EMD Millipore (Lot 4144820).

### 2.2. Instrumentation

Images of the paper-based devices were taken in a light control box (Sanoto MK45 Studio, Shenzhen, China) using a Galaxy S21+ smartphone (Samsung, Suwon, South Korea) or directly scanned (Ricoh IM C3510, Lawrenceville, GA, USA). Smartphone images were used for evaluating the relative performance of assays in the studies to determine the optimal color channel for evaluation and optimal reagent concentrations. The scanner was used in studies to evaluate human serum samples to further minimize errors related to changes in lighting conditions for the more accurate determination of TAC. Solution-based TEAC assay measurements were made using a GeminiXS microplate reader (Molecular Devices, San Jose, CA, USA). Stock solutions and dilutions were measured spectroscopically using an Eppendorf BioSpectrometer^®^ with µCuvette (Hamburg, Germany).

### 2.3. Fabrication of Paper-Based Devices

The paper-based devices used in this work were fabricated using a method developed previously by the Remcho research group [43]. This method uses selective laser ablation to pattern test zones in cellulose-based sheets supported by a waxy backing. Assay chemistry is performed within the patterned test zones. Briefly, a rectangle of Parafilm was pre-cut using an electronic cutter (Quickutz Silhouette SD, Silhouette America, Lindon, UT USA). A Whatman 1 paper of the same dimensions was cut using a CO_2_ laser cutter (VLS 3.50, Universal Laser Systems, Scottsdale, AZ, USA). The parafilm and Whatman 1 paper were aligned and physically bonded using a thermal laminator (Scotch^TM^ TL902A, Maplewood, MN, USA). The circular test zones were designed to be 8 mm in diameter to mimic the dimensions of a well in 96-well plate and were patterned using a 455 nm diode laser (AtomStack A5 laser engraving machine, Shenzhen, China). The number of total test zones varied from 12 to 36 zones per device.

### 2.4. Preparation of Paper-Based TEAC Assay

A total of 40 mM ABTS stock and 70 mM K_2_S_2_O_8_ stock solutions were prepared in ultrapure water. Then, 20 mM and 10 mM dilutions of ABTS and 1, 5, 10, 25, and 50 mM dilutions of K_2_S_2_O_8_ were prepared from stock. A 2.5 mM stock solution of Trolox was prepared in 1X PBS and dissolved via sonication for 1 h. Serial dilutions of 2.0, 1.5, 1.0, 0.5, and 0.25 mM Trolox were prepared from stock. For devices in which human serum was tested, the absorbances of Trolox and ABTS solutions were measured spectroscopically to determine exact concentrations for the more accurate determination of TAC. Trolox was measured at 290 nm (molar extinction coefficient [ε290], 2350 M^−1^ cm^−1^) and ABTS was measured at 420 nm (molar extinction coefficient [ε420], 36,000 M^−1^ cm^−1^).

**Figure 1:**
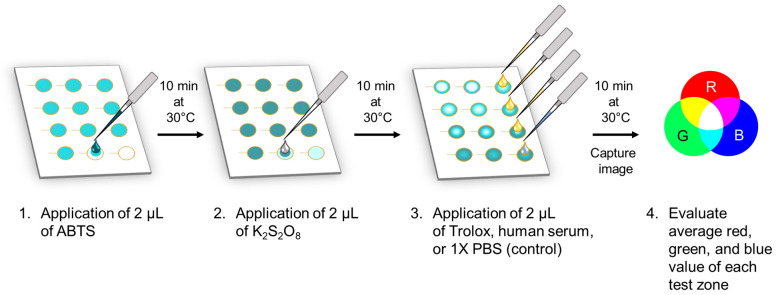
Preparation of paper-based Trolox Equivalent Antioxidant Capacity (TEAC) assays.

As illustrated in Figure 1, 2 µL of ABTS at a chosen concentration and 2 µL K_2_S_2_O_8_ at a chosen concentration were drop-cast sequentially to each test zone. A total of 2 µL of each dilution of Trolox or human serum was then applied to individual test zones in triplicate, with three test zones left untreated with the antioxidant. A 10 min drying step at 30 °C was performed in between the applications of each solution. This temperature was chosen based on a qualitative study comparing reagent-drying at room temperature, 30 °C, and 50 °C. Drying at room temperature resulted in excessively long device preparation times (>1 h) whereas the effects of drying at 50 °C on the reagent stability and TAC determination were unclear. Images of the device were captured after the final drying step.

### 2.5. Colorimetric Analysis of Paper-Based Devices

The average red (R), green (G), blue (B), and combined red–green–blue (RGB) color intensity of the individual test zones in each image was evaluated using ImageJ software version 1.54 (National Institutes of Health, Bethesda, MD). For images that were taken with a smartphone, the images of the paper-based TEAC assay were white-balanced to reduce variations in lighting from image to image before colorimetric analysis. The images of scanned devices were analyzed directly. Standard calibration curves were created by taking the difference in the color intensity of test zones before and after the application of Trolox, which is analogous to the approach used to evaluate solution-based TEAC assays [34,44]. The degree of decolorization in the assay zones was calculated, where I_f_ was the final average color intensity after the application of Trolox standards or sample and I_c_ was the initial average color intensity of the control test zones (Equation (1)). I_c_ was determined by evaluating the test zones untreated by Trolox, samples, or blanks.

Δ Color intensity = I_f_ − I_c_, (1)

This value was further adjusted to account for the decolorization attributed to the application reagents (Equation (2)). This value was represented by the average color intensity of the test zone after application of the blank control, 1X PBS, (I_b_).

Δ Color intensity (adjusted) = Δ Color intensity − I_b_, (2)

The adjusted change in color intensity is used in standard curves and sample evaluations by µPADs throughout this study.

### 2.6. Solution-Based TEAC Assay

A standard, solution-based TEAC assay was run to validate the results acquired using the paper-based TEAC assay. The preparation and the chosen reagent concentrations of were based upon those in pre-made TEAC assay kits used in clinical settings [45]. 50X ABTS^•+^ was prepared by combining 5 mg of ABTS in 1.170 mL of water with 130 µL 2.5 mM K_2_S_2_O_8_ in water. The solution was prepared in an amber vial and stored at 4 °C overnight for complete radical cation formation. On the day of use, the ABTS^•+^ solution was diluted 50-fold with 1X PBS to a final concentration of 0.15 mM. Dilutions of 250, 200, 150, 100, 50, and 25 µM Trolox in PBS were prepared from 2.5 mM Trolox stock and measured spectroscopically at 290 nm to determine the actual concentrations. A 96-well microplate was pre-loaded with 25 µL of Trolox standards in triplicate, with 1X PBS serving as a blank control. A total of 150 µL of the diluted ABTS^•+^ solution was added to each well using a multi-channel autopipette to minimize delay, as the reaction is time-sensitive. The microplate was placed on a shaker at 200 rpm and the absorbance of each well was measured at 734 nm after 5 min using a microplate reader.

### 2.7. Preparation and Use of Human Serum Samples

Frozen normal human serum was used as a representative real sample. A new aliquot of frozen serum was thawed on each day of evaluation. Human serum was diluted in 1X PBS at 15%, 20%, 25%, and 50% *v*/*v* for paper-based devices and at 5.0, 7.5, and 10.0% *v*/*v* for solution-based experiments.

### 2.8. Limit of Detection and Statistical Testing

For both paper-based devices and solution-based devices, the limit of detection (LOD) was calculated as the quotient of the 3× standard deviation of the lowest concentration of the tested standard and the slope of the calibration curve, per the IUPAC definition of LOD.

A two-sided paired t-test was used to determine whether the differences in mean TAC values acquired from paper-based and solution-based TEAC assays were statistically significant.

## 3. Results

### 3.1. Determining the Optimal Color Channel for Evaluation of of ABTS^●+^ Decolorization

**Figure 2:**
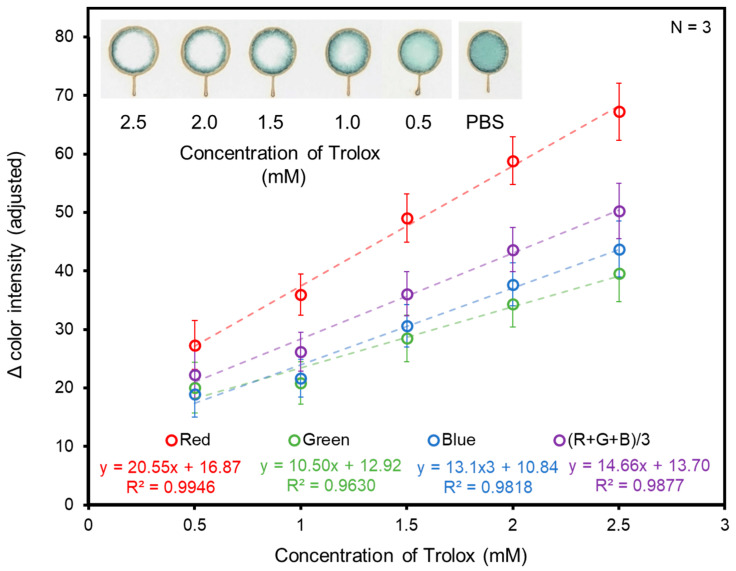
Paper-based TEAC assay response to standards of Trolox for red, green, blue, and combined RGB color channels. N = 3 for all points.

The responses of the three individual color channels (R, G, B) and the average intensity of all three color channels (RGB) were evaluated to determine the color channel for optimal sensitivity and linearity. The paper-based TEAC device used in these experiments was treated with 40 mM ABTS and 5 mM K_2_S_2_O_8_ and standards of Trolox ranging between 0.5 and 2.5 mM. The difference in color intensity before and after the application of Trolox was determined (Figure 2).

Although a change in the blue-green color was visible on the device, the red color channel was chosen as it showed the greatest change in color intensity after the application of Trolox. The red color channel exhibited the greatest change in color intensity because its initial intensity was less than the initial intensities of the green and blue color channels prior to the application of Trolox. As decolorization occurred with increasing concentrations of Trolox, all three color channels trended towards white (i.e., the maximum RGB color value, 255). Subsequently, a greater change in red color intensity occurred to reach 255. An additional advantage of choosing the red color channel for future analyses was that it exhibited the greatest degree of linearity (R^2^ = 0.995) as compared to all other color channels. As such, future test zones on devices were evaluated for the change in red color intensity before and after the application of an antioxidant.

### 3.2. Optimization of Reagent Concentrations for Construction of a Calibration Curve

The determination of the TAC of biological samples on the µPAD is directly achieved through the standard curve formed via colorimetric analysis of Trolox standards. Trolox is an analog of α-tocopherol, a natural biological antioxidant. It was of the utmost importance to produce a standard curve with the optimal sensitivity and dynamic range to accurately determine TAC levels in real samples.

Ensuring the correct balance between the concentration of ABTS and K_2_S_2_O_8_ applied to each test zone is necessary, as too little of either can result in undetectable color development (i.e., inadequate formation of ABTS^●+^) whereas too much of either can result in undetectable decolorization after the application of the antioxidant (i.e., excess formation of ABTS^●+^). The concentration of K_2_S_2_O_8_ and ABTS applied to the test zones was selected to yield optimal signal sensitivity and the greatest dynamic range. To determine the optimal concentration for each respective assay component, 15 combinations of ABTS and K_2_S_2_O_8_ concentrations were tested using 10, 20, and 40 mM ABTS and 1, 5, 10, 25, and 50 mM K_2_S_2_O_8_. The evaluation of signal sensitivity and dynamic range was based upon standard curves created via the application of 0–2.5 mM Trolox standards to paper-based devices treated with varying combinations of ABTS and K_2_S_2_O_8_. A total of 2.5 mM Trolox was selected to ensure maximum solubility in 1X PBS. The 1X PBS served as a blank control because the decolorization of assay zones occurs on paper-based devices with the application of solution, regardless of the antioxidant content. Accounting for this decolorization facilitates discernment between decolorization attributed to antioxidant capacity as compared to decolorization caused by the physical application of solution—a critically important distinction that, left unaccounted for, would lead to erroneous reporting.

**Figure 3:**
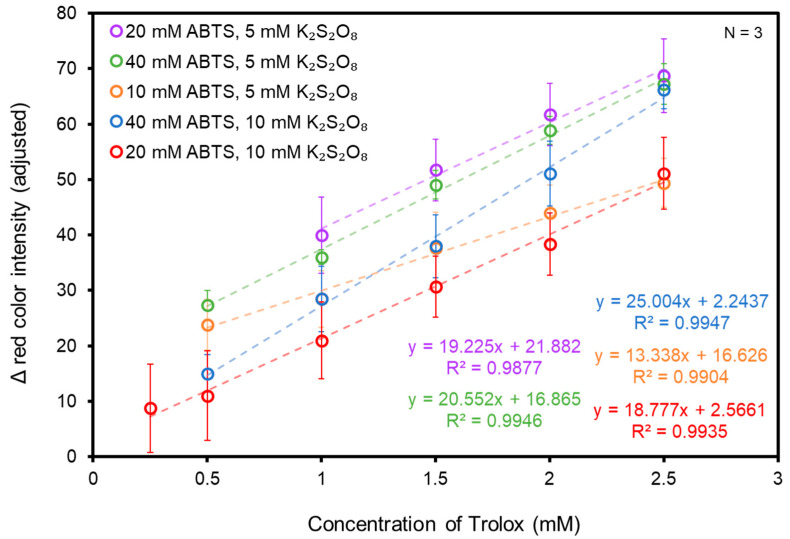
Dynamic range and sensitivity of the paper-based TEAC assay at various ABTS and K_2_S_2_O_8_ concentrations.

Of the concentrations of ABTS that were tested, linear trends were not observed with the use of 1 mM or 50 mM K_2_S_2_O_8_ and were therefore not included in Table 1. We hypothesize that 1 mM results in inadequate color development. Subsequently, when the standards of Trolox are applied, complete decolorization occurs regardless of concentration. At the other extreme, the use of 50 mM K_2_S_2_O_8_ results in excessive color development regardless of the concentration of ABTS. The remaining combinations of K_2_S_2_O_8_ and ABTS showed a decreasing trend of decolorization, but not a consistent change in red color intensity, making those specific combinations of reagent concentrations unsuitable for evaluating real samples. The reagent combinations with a linear working range of at least 4 points and with R^2^ values greater than 0.98 are plotted in Figure 3.

Devices treated with 40 mM ABTS and 10 mM K_2_S_2_O_8_ yielded the greatest signal sensitivity, as indicated by a slope of 25.0 intensity units/mM of Trolox. Although the combination of 20 mM ABTS and 10 mM K_2_S_2_O_8_ was less sensitive than other combinations, it yielded the largest dynamic range. We initially had concerns regarding whether human serum had sufficient TAC for detection with the paper-based device and therefore preferentially chose the conditions with the largest dynamic range over the conditions with higher sensitivity. In a practical sense, this combination of concentrations also had an advantage in minimizing reagent consumption.

Although the degree of color development and decolorization depends on the concentrations of ABTS and K_2_S_2_O_8_ deposited, it was also observed during this investigation that ABTS^●+^ formation occurs rapidly after the deposition and drying of K_2_S_2_O_8_ on the paper-based device, regardless of reagent concentrations. This allows the device to be used immediately for the analysis of samples after a short treatment with ABTS and K_2_S_2_O_8_. In contrast, solution-based TEAC assays require the overnight incubation of ABTS and K_2_S_2_O_8_ to ensure the complete formation of ABTS^●+^ before use. This is a significant advantage of the paper-based TEAC platform; the preparation required before use is reduced in comparison with its solution-based counterpart.

### 3.3. Validation of Paper-Based Device’s Performance Using Human Serum Samples

**Figure 4:**
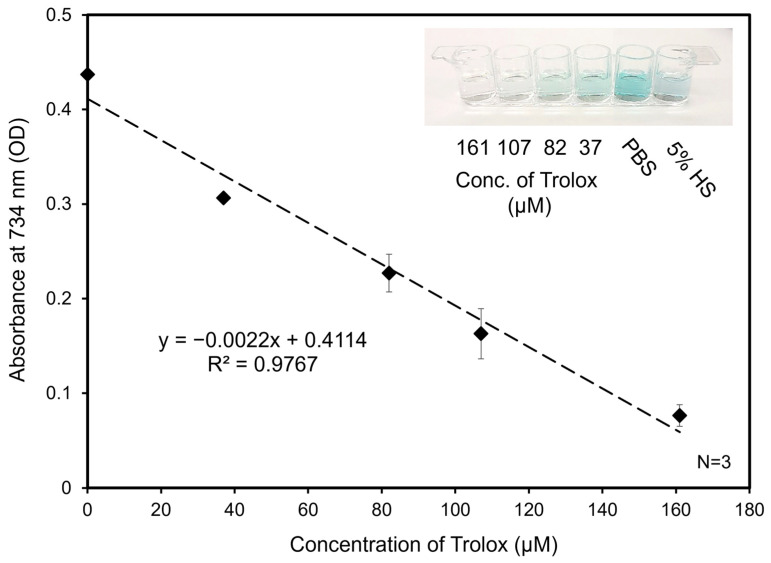
Standard curve from solution-based TEAC assay. Absorbance was acquired at 734 nm for Trolox standards at 161, 107, 82, and 37 µM after 5 min of reacting with ABTS^•+^. Trolox standards were measured spectroscopically prior to use in the TEAC assay to determine concentrations. 1X PBS was used as a blank control and 5% human serum in PBS was used as a sample. The absorbances of all solutions were evaluated in triplicate.

Solution-based TEAC assay experiments were carried out for a performance comparison to demonstrate the validity of the results acquired from the paper-based TEAC assay devices. A solution-based TEAC assay was formulated based on those used in clinical lab settings [45]. Figure 4 is an example of a standard curve generated by Trolox of between 0 and 161 µM. Initially, 25, 50, 100, 150, 200, and 250 µM Trolox standards were prepared from 2.5 mM Trolox stock. However, after the absorbance of the diluted standards at 290 nm was measured, only four standards were used. The absorbances of the two most diluted samples were only negligibly greater than that of the blanks; therefore, they were not included in the standard curve as their actual concentrations could not be determined. The use of 1X PBS as a control and the effects of 5.0%, 7.5%, and 10% *v*/*v* human serum in 1X PBS were evaluated using the calibration curve.

The average TEAC values of 5.0%, 7.5%, and 10.0% human serum in 1X PBS was determined to be 2.06 ± 0.13, 1.72 ± 0.03, and 1.57 ± 0.03 mM, respectively, as measured at 734 nm. These values represent the mmol equivalents of Trolox/L, which is analogous to the TAC of the original, undiluted serum sample. Despite originating from the same sample, the serum dilutions led to different calculated TAC values. This result is expected; it was previously shown that dilutions of serum result in a non-linear response, which may be attributed to the kinetics of the reaction at different antioxidant concentrations [31]. This is accepted as an inherent characteristic of the TEAC assay regardless of the platform the assay is performed on. Of note, these values are slightly greater than the average TAC values of normal human serum, which typically range from 0.513 to 1.50 mM [26,31,46,47]. If deproteination has not been performed, the TAC value can be elevated, as is the case in this study [48].

**Figure 5:**
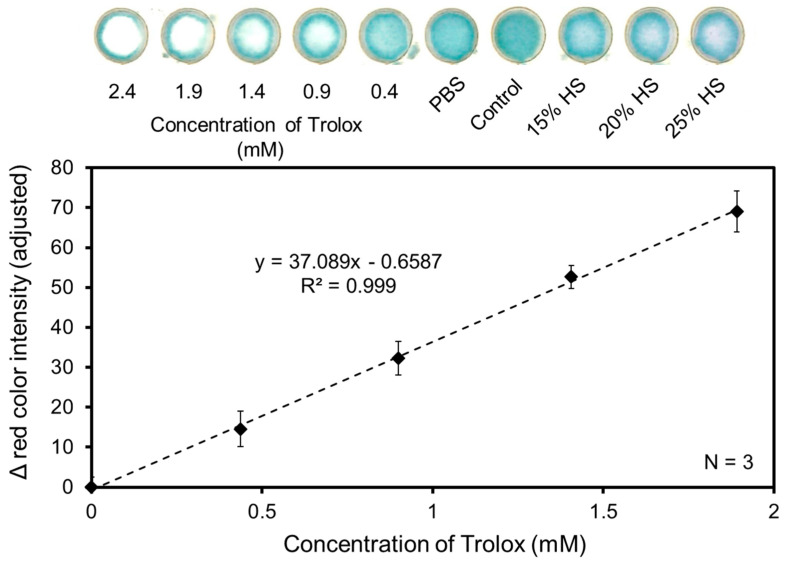
Standard curve from the paper-based TEAC assay. Trolox standards were measured spectroscopically prior to use in the TEAC assay to determine concentrations. 1X PBS was used as a blank control and 15%, 20%, and 25% human serum in PBS were used as samples. The 50% and 100% human serum is not shown. Standards, samples, and controls were analyzed using colorimetric methods in triplicate.

The previously established concentrations of ABTS and K_2_S_2_O_8_ (20 mM and 10 mM, respectively) were used to test the antioxidant capacity of human serum samples on the µPAD. A new standard curve was generated using 2.4, 1.9, 1.4, 0.9, and 0.4 mM Trolox (Figure 5). As with the solution-based TEAC assay, the exact concentration of the Trolox standards were established spectroscopically to achieve an accurate TAC determination of human serum. Test zones without the additional application of antioxidant or PBS were designated as a control to determine the initial average color intensity of test zones before decolorization via the application of Trolox (I_c_). Dilutions of 15%, 20%, 25%, 50%, and 100% *v*/*v* human serum in 1X PBS were evaluated. The bleeding of assay zones occurs due to the saturation of these areas with previously applied reagents. Some bleeding of reagents outside of the test zones was observed but this had no major effects on the linearity of the standard curve (R^2^ = 0.99). Additionally, the % relative standard deviation in color intensity between test zones treated with the same concentrations of antioxidant or sample was within 5%.

**Figure 6:**
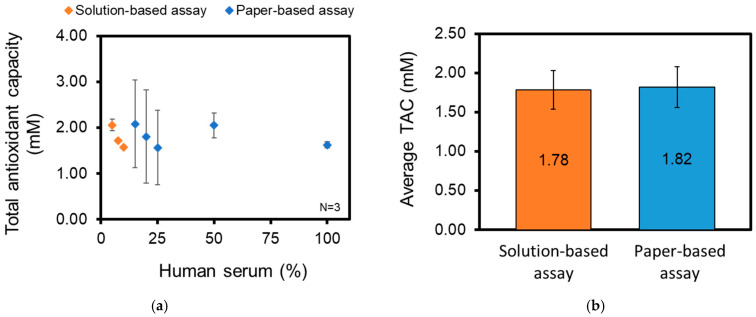
(**a**) Comparison of Total Antioxidant Capacity (TAC) determined using a solution-based TEAC assay with absorbances acquired at 734 nm (orange marker) and a paper-based TEAC assay with adjusted values (blue marker) at various dilutions of human serum. N = 3 for all solution based-assays and paper-based assays. (**b**) Average TAC of human serum sample, as determined by a solution-based TEAC assay and a paper-based TEAC assay using multiple dilutions of human serum. N = 9 for solution-based assays; N = 15 for paper-based assays.

The average TAC values of 15%, 20%, 25%, 50%, and 100% human serum in 1X PBS were determined to be 2.08 ± 0.96, 1.81 ± 1.01, 1.57 ± 0.81, 2.05 ± 0.27, and 1.62 ± 0.07 mM respectively (Figure 6a). 

The average calculated TAC was 1.78 ± 0.25 and 1.82 ± 0.26 mM, as determined from the solution-based assay and the paper-based assay, respectively (Figure 6b). There was a 0.04 mM difference between the solution-based and paper-based assay, which is well within the one standard deviation of the solution-based assay. The paired *t*-test had a *p*-value of 0.816, which suggests that the difference in the means was not statistically significant. This further supports the claim that the paper-based device performs with reasonable accuracy when compared to the solution-based device.

The discrepancy in the average TAC values between the two platforms can be attributed to the formation of a purple product that causes interference in the measured changes in the color intensity of red. It has been proposed that the purple adduct is the result of tyrosyl residues from serum proteins interacting with ABTS^●+^ [49]. This hypothesis was further confirmed in our qualitative selectivity studies using bovine serum albumin standards (Figure S4). The decolorization and development of purple products due to the albumin standards in assay zones was observed. The product increases the signal in the red color channel for the colorimetric detection of the paper-based TEAC devices and subsequently contributes to an elevated TEAC value. The purple product is also present in solution-based TEAC assays but does not cause interference at 734 nm. The difference between solution and paper-based TEAC values may also be due to the formation of dityrosine, which also contributes to an elevated TEAC value [49]. This would also be the case with the solution-based assay, but possibly to a lesser extent than is observed with the paper-based device. Although alternative TAC assays do not exhibit colorimetric interference due to serum proteins, they are unsuitable for paper-based platforms for reasons such as their temperature dependence and lack of compatibility with the evaluated proteins [32,33,42,50].

It is apparent that paper-based devices have a larger range of variability than solution-based analyses for individual dilutions of human serum. The batch-to-batch variability of the fabricated µPADs was not directly investigated, but the consistency in the Trolox standard curves acquired across multiple devices seems to imply that the fabrication method is not a concerning factor in the variability of assay performance. In contrast, the bleeding of reagents out of test zones contributes to variations in the results. Increasing the drying temperature to 50 °C would eliminate reagent bleed and reduce the standard deviation for individual dilutions of samples (Figure S3). However, further studies on the effects of drying temperature on ABTS^•+^ stability and the accuracy of the subsequent TAC determination need to be performed. Another contributing factor to device variability is sample dilution. Variations in TAC values are magnified after accounting for dilution factors when determining the TAC of human serum using the standard curve. Subsequently, the range of error for samples decreases with increases in the concentration sample concentration. While the TAC of 100% normal human serum is within the linear range of the standard curve, a more accurate result can be achieved by averaging the TAC values acquired from multiple dilutions of human serum because the interference arising from the formation of the purple byproduct is not as prevalent at lower sample concentrations. Accurate results are achieved through balancing the variability observed at lower sample concentrations with the interference present at higher sample concentrations.

The limit of detection (LOD) for the paper-based assay was determined to be 0.20 mM, while the LOD for the solution-based assay is 0.0053 mM. Although there is a significant difference in the LOD between platforms, both approaches are adequate to monitor health conditions that result in decreased or elevated levels of TAC compared to normal serum levels. Solution-based TEAC assays require the dilution of samples for evaluation, regardless of the individual’s health status. The dilution of samples may or may not be necessary for evaluation with paper-based TEAC assays. As stated previously, 0.513 to 1.50 mM is the range of TAC values for normal human serum. It is unlikely for the TAC value to be below the LOD of the paper-based assay even in conditions where attenuated levels of TAC are expected. In the case of health conditions that cause an elevated TAC, such as sepsis, the sample could be diluted to within the linear working range of the paper-based assay [51].

### 3.4. Stability Studies

**Figure 7:**
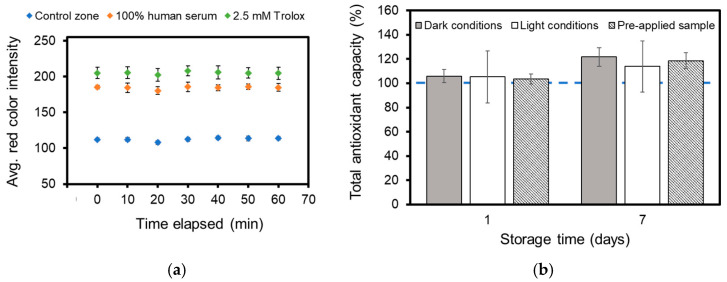
(**a**) Stability of paper-based TEAC assays one hour after the application of Trolox or a human serum sample. N = 3 for all evaluated test zones. (**b**) Storage stability of paper-based TEAC assay when stored in the dark in the refrigerator and in the light at room temperature. The stability was demonstrated as the % change in TAC value, with 100% being the TAC value determined using freshly prepared devices for the analysis of 15% human serum in 1X PBS. Additional devices with pre-applied reagent and samples were also re-evaluated 7 days later. N = 3 for all conditions.

The response stability of the µPADs was evaluated after use. Control zones without antioxidant application, test zones with the application of human serum, and test zones with the application of 2.5 mM Trolox were evaluated in 10 min increments over a 1 h period to investigate any changes in red color intensity over time (Figure 7a). Control zones had changes in red color intensity of less than 2.12 units, corresponding to a 1.88% change in intensity. Similarly, test zones where human serum or Trolox were applied only showed a 1.01 and 0.81% change in red color intensity, respectively. In contrast, solution-based TEAC assays begin to decolorize and continue to decolorize once the antioxidant has been added to the ABTS^•+^ solution. As such, the sample must be evaluated after a short period of time, typically 5 min. It is an advantage that paper-based TEAC assays can be used and evaluated over a long time period without major changes in response.

Additionally, the storage stability of pre-made µPADs was investigated. The test zones were treated with ABTS and K_2_S_2_O_8_, as described in Section 2.4. The µPADs evaluated over the course of several days were stored in two environments. One set of devices was stored at room temperature in the light; the other set of devices was stored at 4 °C in foil packets to protect them from light. Devices were evaluated using Trolox and 15% human serum after storage for 1 day or 1 week. In addition, the storage stability of used µPADs was evaluated. Human serum samples were pre-applied to the device and stored at room temperature before being evaluated after 1 day or 1 week.

Devices stored over 7 days had elevated TAC values regardless of storage conditions. Stored devices demonstrated an increase in perceived TAC value of 13.19% and 21.7% for light and dark conditions, respectively (Figure 7b). This would be considered a significant increase in TAC level when using the TAC for health monitoring. It is likely that oxidation of the applied reagents occurred because these devices were not stored in the absence of oxygen. Interestingly, the devices in which assay reagents, samples, and standards were pre-applied on the same day, but evaluated a week later, showed a similar increase in TAC value. Devices evaluated after 1 day of storage, regardless of storage conditions, were within 6% of the accuracy of freshly prepared devices. The devices with a pre-applied sample showed an increase in TAC value of 3.67%. This may suggest that devices can be stored for assessment a day later if all reagents and samples are applied within the same day. The slight increase in perceived TAC value can be attributed to oxidation, light-bleaching, or a combination of both. The general recommendation is that the devices are used within one day of preparation for the most accurate results. Despite the lack of long-term stability, paper-based devices have greater stability than solution-based TEAC assays, which must be evaluated within minutes of the sample’s addition.

## 4. Discussion

Portable platforms for TAC determination have been developed but are largely focused on measuring TAC in food or environmental samples. An accurate, simple, and portable method for measuring TAC would be of great benefit for individuals who want to monitor their TAC levels in response to treatment over time. The proposed µPAD has potential for use in point-of-care settings when combined with additional blood-separation devices. Capillary and venous plasma were previously found to have significantly similar TAC values [52]. As such, end-users could use a lancet to collect finger-prick blood samples for TAC evaluation. The whole blood samples would be separated using point-of-care microfluidics or centrifuges. Metered volume applicators, such as fixed-volume 2-µL capillaries, would be utilized for the accurate liquid handling of reagents and samples in order to prepare and use the devices. Images of the used devices could easily be evaluated by experienced personnel via telehealth or mail-in health diagnostics, or in real-time by automated programs. The developed paper-based TEAC assay could be one test in a suite of point-of-care tests that an individual uses and submits for professional evaluation. An automated instrument that is capable of performing sample separation, liquid handling for µPAD preparation and use, and detection would be ideal to minimize human handling operations and associated errors but is beyond the scope of this work. In its current state, the proposed paper-based TEAC assay may better serve as an alternative approach to solution-based TEAC assays in clinical settings based on the ease of preparation of these devices and the prolonged stability of the assay compared to standard solution-based TEAC assays.

The TEAC µPAD features a large working range suitable for monitoring changes in TAC in serum for multiple health conditions, such as increases in serum TAC following treatment for major depressive disorder [18]. The LOD is adequate for evaluating attenuated levels of serum TAC associated with conditions like type 2 diabetes [15,53]. It is important to note that TAC values are confounded by a variety of external influences, most notably diet, environmental, and behavioral factors. As such, serum TAC alone cannot be used as a diagnostic marker but can be used in tandem with analyses of other well-established biomarkers of disease to provide a more comprehensive diagnosis of health status. In addition, complex comorbidities are potential confounding factors in accurate TAC determination; therefore, an assessment of additional biomarkers could inform end users of the reliability of their TAC results. The µPAD developed here could be used in conjunction with other assays adapted to the same platform or with separate point-of-care assays. For example, the total protein concentration could be determined using this platform via tetrabromophenol blue assay chemistry (as demonstrated in Figures S1 and S2). This could be relevant to evaluations of the risk or prognosis of conditions such as coronary heart disease, where decreased levels of serum albumin and TAC are observed [10,11,54,55,56]. The combination of TAC and total protein concentration analyses on the same platform leads to more robust interpretations of oxidative stress conditions and diagnoses. Further studies that incorporate additional assays to evaluate an array of biomarkers depending on the condition or disease would be a natural progression of this work.

## 5. Conclusions

In this work, we developed, validated, and applied a paper-based TEAC assay that is compatible with the evaluation of human serum samples. To our knowledge, this is the first paper-based TEAC assay used in TAC analysis with human serum. A total of 20 mM ABTS and 10 mM K_2_S_2_O_8_ were established as the optimal reagent concentrations for the adaptation of a TEAC assay to a paper-based platform based on the working range (0.44–2.4 mM). The average TAC value of a normal human serum sample evaluated using the µPAD (1.82 ± 0.24 mM) was comparable to the average value acquired from a solution-based TEAC assay (1.78 ± 0.25 mM).

The adaptation of this assay to a portable platform has multiple advantages: it can be fabricated for high-throughput analyses, requires 10-fold less reagent consumption, and reduces preparation time from multiple days to less than 2 h as compared to standard solution-based assays. Additionally, the µPADs can be prepared and used, and samples evaluated, within one day, in contrast with solution-based assays which require spectroscopic evaluation minutes after the addition of the sample. The LOD of the device, 0.20 mM, is more than adequate for quantitative analyses of normal human serum samples and samples with attenuated levels of TAC. Although the µPAD introduced in this work does not outperform solution-based TEAC assays in terms of sensitivity or LOD, this approach is amenable to point-of-care settings, with a comparable accuracy to the standard spectroscopic techniques and with a working range that is relevant to conditions where TAC is monitored. Ultimately, this work expands the field of point-of-care health by introducing an accessible method for measuring TAC in human serum.

## Figures and Tables

**Table 1:** Dynamic range and sensitivity of the various combinations of 2,2’-azinobis(3-ethylbenzothiazoline-6-sulfonic acid) (ABTS) and potassium persulfate (K_2_S_2_O_8_) that elicited a linear response using the TEAC µPAD.

Reagent Combinations	Slope	Range	R^2^
10 mM ABTS			
5 mM K_2_S_2_O_8_	13.30	0.5–2.5 mM	0.99
20 mM ABTS			
5 mM K_2_S_2_O_8_	19.23	1.0–2.5 mM	0.99
10 mM K_2_S_2_O_8_	18.78	0.25–2.5 mM	0.99
40 mM ABTS			
5 mM K_2_S_2_O_8_	20.55	0.5–2.5 mM	0.99
10 mM K_2_S_2_O_8_	25.00	0.5–2.5 mM	1.00
25 mM K_2_S_2_O_8_	11.47	0.5–2.0 mM	0.98

## Data Availability

The original contributions presented in the study are included in the article; further inquiries can be directed to the corresponding author.

## Supplementary Materials

The following supporting information can be downloaded at: https://www.mdpi.com/article/10.3390/bios14110559/s1, Figure S1: Paper-based devices with TBPB assay chemistry for the determination of protein concentration in human serum; Figure S2: Standard curve from paper-based TBPB assay and comparison of average total protein concentrations; Figure S3: Uncropped images of TEAC assay devices dried at 50 °C and 30 °C; Figure S4: Uncropped image of TEAC assay used to evaluate BSA standards.
