# Supplementary material for: A Paper-Based Assay for the Determination of Total Antioxidant Capacity in Human Serum Samples
Source: Biosensors (Basel). 2024 Nov 18;14(11):559. doi: 10.3390/bios14110559 (PMC11591649; doi:10.3390/bios14110559)
Supplement: Supplementary file 1 [file biosensors-14-00559-s001.zip › biosensors-3281385-supplementary.pdf]

biosensors

MDPI

*Supplementary Material*

# A Paper-Based Assay for the Determination of Total Antioxidant Capacity in Human Serum Samples

**Michelle T. Tran, Sophia V. Gomez, Vera Alenicheva and Vincent T. Remcho**

## S1. Experimental method of adapting tetrabromophenol blue assay chemistry to a paper-based device

Tetrabromophenol blue (TBPB) is used to determine total protein concentration in human serum. To prepare devices, 2 µL of 250 mM citrate buffer, pH 4 and 1.5 µL of 5 mM TBPB in 95% ethanol were applied sequentially to the test zones with a 10 minute drying step at 30°C in-between applications of reagents. 2.5 µL bovine serum albumin (BSA) standards, 1X PBS, or dilutions of human serum samples in 1X were then applied.

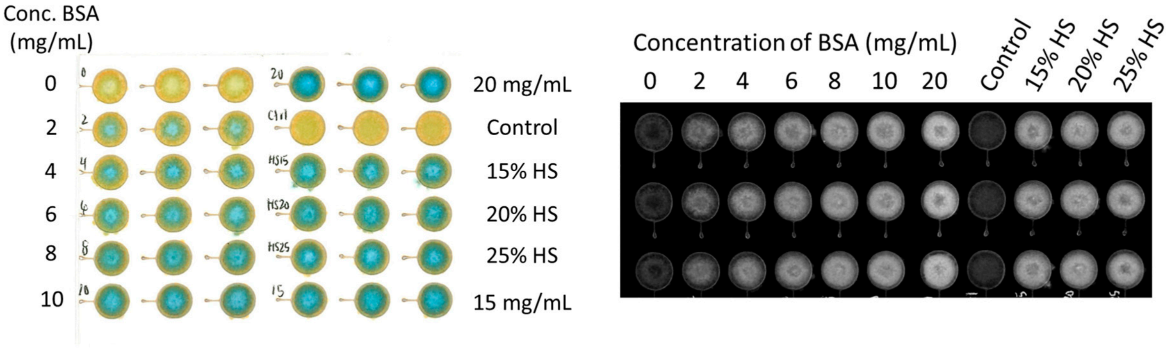


(A) (B)

**Figure S1.** Paper-based devices with TBPB assay chemistry for determination of protein concentration in human serum. 1X PBS was applied for 0 mg/mL concentration of BSA. Control was when no additional sample or standard was applied. (A) Original paper-based devices with 0-20 mg/mL BSA in 1X PBS applied and human serum samples applied. (B) Image-processed assays for evaluation. 15 mg/mL BSA not shown, but was evaluated and used.

Device images were processed in ImageJ by first inverting the image and then splitting the image into red, green, and blue color channels. Only the red color channel was evaluated for average intensity of test zones.

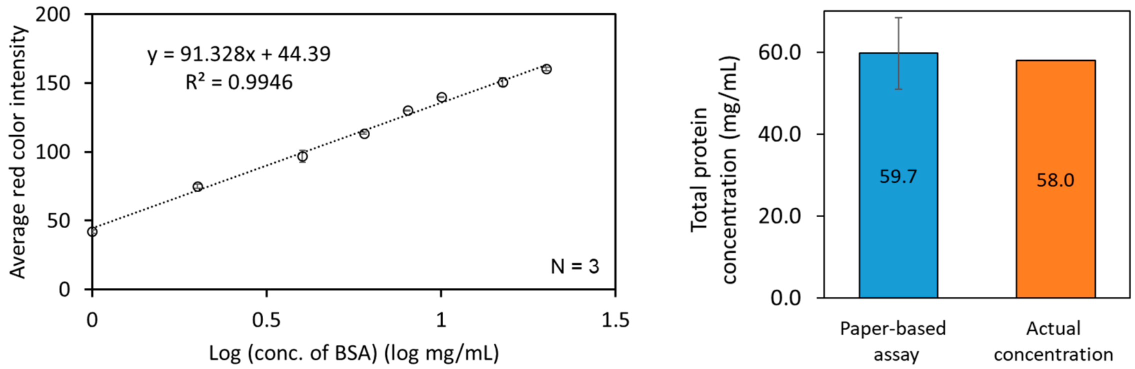


(a) (b)

**Figure S2.** (a) Standard curve from paper-based TBPB assay. Average red color intensity values were determined from image-processed assays. (b) Comparison of total protein concentration determined using the paper-based assay and from the specification sheet provided for the human serum samples. Average was from evaluating 15, 20, and 25% human serum in 1X PBS.

The total protein concentration of human serum as determined by the µPAD was 59.7 ± 8.69 mg/mL. The slight difference in total protein concentration listed on the specification sheet for the human serum sample and as determined by the µPAD may be due to using BSA standards rather than human serum albumin standards.

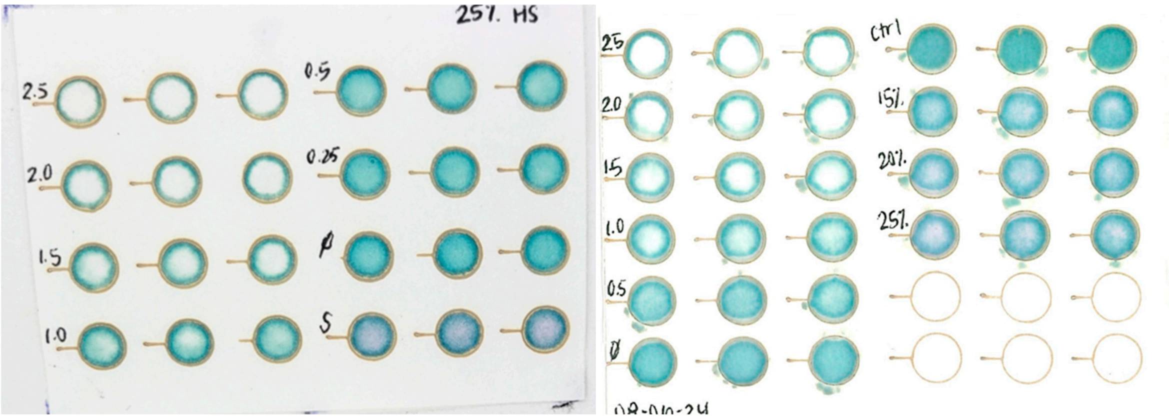


(A) (B)

**Figure S3.** Uncropped images of TEAC assay devices dried at (A) 50°C and (B) 30°C in-between application of reagents and sample. Image of device dried at 50ºC was taken with a smartphone and have no apparent bleeding from zones. The device dried at 30ºC was scanned.

## S2. Selectivity of the TEAC assay

Standards of BSA prepared in 1X PBS were tested on the TEAC assay device to confirm that decolorization and purple product formation was due to the reaction of $ABTS^{\bullet +}$ with the serum albumin. An albumin-based standard was chosen because it is the most abundant source of antioxidants in blood serum. We observed decolorization and formation of purple products with these standards, corresponding to what was observed in evaluating human serum samples.

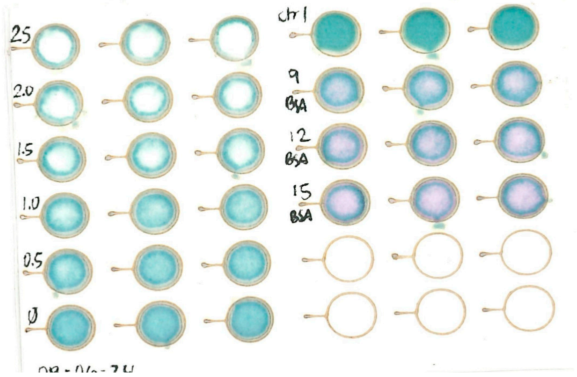


**Figure S4.** Uncropped image of TEAC assay device used to evaluate 9, 12, and 15 mg/mL BSA standards.
